# ﻿Morpho-molecular characterisation of *Arecophila*, with *A.australis* and *A.clypeata* sp. nov. and *A.miscanthi* comb. nov.

**DOI:** 10.3897/mycokeys.88.79475

**Published:** 2022-04-13

**Authors:** Qi Rui Li, Xu Zhang, Yan Lin, Milan C. Samarakoon, Kevin David Hyde, Xiang Chun Shen, Wan Qing Liao, Anuruddha Karunarathna, Si Han Long, Ying Qian Kang, Ji Chuan Kang

**Affiliations:** 1 The Engineering and Research Center for Southwest Bio-Pharmaceutical Resources of National Education Ministry of China, Guizhou University, Guizhou, China; 2 The High Efficacy Application of Natural Medicinal Resources Engineering Center of Guizhou Province (The Key Laboratory of Optimal Utilization of Natural Medicine Resources), School of Pharmaceutical Sciences, Guizhou Medical University, University Town, Guian New District, Guizhou, China; 3 Department of Entomology and Plant Pathology, Faculty of Agriculture, Chiang Mai University, Chiang Mai 50200, Thailand; 4 Center of Excellence in Fungal Research, Mae Fah Luang University, Chiang Rai 57100, Thailand; 5 Shanghai Key Laboratory of Molecular Medical Mycology, Department of Dermatology and Venereology, Changzheng Hospital, Shanghai, China; 6 Department of Microbiology, Guizhou Medical University, University Town, Guian New District, Guizhou, China

**Keywords:** *Cainiaceae*, gramineous plants, phylogeny, taxonomy

## Abstract

Three arecophila-like fungal samples were collected on dead culms of gramineous plants in China. Morphological studies of our new collections and the herbarium specimen of *Arecophilagulubiicola* (generic type) were conducted and the morphological affinity of our new collections with *Arecophila* was confirmed. Maximum likelihood and Bayesian analyses using combined ITS, LSU, *rpb*2 and β-tubulin data from our collections revealed the phylogeny of *Cainiaceae*. The monospecific genus *Alishanica* (type species *Al.miscanthi*), which had been accepted in *Cainiaceae*, is revisited and synonymised under *Arecophila*. Based on morphology and phylogeny, *Arecophilaaustralis* sp. nov. and *A.clypeata* sp. nov. are introduced as new species, while *A.miscanthi* is a new record for China. All the new collections are illustrated and described.

## ﻿Introduction

The current study is a part of a series of papers on Xylariales (Sordariomycetes) from China (Long et al. 2019; Xie et al. 2019, 2020; Pi et al. 2020). *Arecophila* K.D. Hyde, which is typified by *A.gulubiicola* K.D. Hyde, was introduced by Hyde (1996) with five species. *Arecophila* is characterised by immersed, subglobose to lenticular ascomata, peridium with *textura angularis* cells, non- or poorly-developed clypeus, asci with a wedge-shaped, apical ring, J+ in Melzer’s reagent and 2-celled, brown ascospores with wall striations, surrounded by a mucilaginous sheath. Thanks to subsequently undertaken morphological studies of holotypes, several species have been transferred to *Arecophila* from genera such as *Amphisphaeria* Ces. & De Not., *Cainia* Arx & E. Müll., *Didymosphaeria* Fuckel and *Schizostoma* Ehrenb. ex Lév. (Hyde 1996; Umali et al. 1999; Wang et al. 2004).

Currently, there are 15 *Arecophila* epithets in Index Fungorum (http://www.indexfungorum.org/Names/Names.asp, May 2021), which have been introduced, based on morphology and lack sequence data (e.g. Hyde 1996; Umali et al. 1999; Wang et al. 2004). After searching for *Arecophila* in NCBI, there were only five hits of LSU, SSU and metagenomic sequences of *A.bambusae* and *Arecophila* sp. HKUCC 6487 in GenBank.

*Arecophila* was introduced as a genus of *Amphisphaeriaceae* (Hyde 1996), based on its unitunicate, cylindrical asci with a J+ apical ring and brown, 2-celled ascospores. Kang et al. (1999) reviewed the genus and accepted it in *Cainiaceae* and the occurrence on monocotyledons (palms and bamboo). The single and combined molecular analyses of LSU and SSU genes resulted in *Arecophila* grouping with *Cainia* in *Xylariales* (Smith et al. 2003). Based on analyses of partial LSU gene sequences, the generic placement of *Arecophila* within the *Cainiaceae* has been verified (Jeewon et al. 2003; Senanayake et al. 2015; Hyde et al. 2020; Wijayawardene et al. 2020). However, the available molecular data do not provide strong evidence of the phylogenetic affinity of *Arecophila* and related taxa.

During our continuous collecting of xylarialean taxa in China, we found some specimens that share a morphology resembling *Arecophila*. In this paper, two new species and a new record of *Arecophila* are provided with descriptions and illustrations. Furthermore, *Alishanica* is synonymised under *Arecophila*, based on morphology and phylogeny.

## ﻿Materials and methods

### ﻿Collection, isolation and morphology

Fresh samples were collected in Guizhou and Yunnan Provinces in China during the rainy season and taken to the laboratory in paper bags. Single-spore isolations were obtained following the method described in Chomnunti et al. (2014). The cultures on potato dextrose agar (**PDA**) were transferred to 2 ml screw cap centrifuge tubes filled with 10% glycerol and sterile water to deposit at –20 °C and 4 °C, respectively. Herbarium materials were deposited at the Herbarium of Guizhou Agricultural College (**GACP**) and the Herbarium of Guizhou University (**GZUH**). Cultures were deposited at the Culture Collection of Guizhou University (**GZUCC**).

The morphological examination of fresh and herbarium specimens was carried out as described by Hyde (1996). Macro-morphological characters were examined and photographed using a digital camera (Canon 700D) fitted to the Olympus SZ61 stereomicroscope. Materials mounted in water, Melzer’s reagent and Indian ink were examined. At least 30 ascospores, 30 asci and 20 apical rings were measured for each taxa with Tarosoft (R) Image Frame Work (v. 0.9.0.7) and photographed using a digital camera (Nikon 700D) fitted to a light microscope (Nikon Ni).

### ﻿DNA extraction, polymerase chain reaction (PCR) amplification and sequencing

Total genomic DNA was extracted from fresh mycelium scraped off from pure cultures with the BIOMIGA fungus genomic DNA extraction kit (GD2416) (Wijayawardene et al. 2013) following the manufacturer’s instructions. Primers, LR0R/LR5 (Vilgalys and Hester 1990), ITS4/ITS5 (White et al. 1990), RPB2-5F/RPB2-7cR (Liu et al. 1999), Bt2a/Bt2b and ACT-512F/ACT-783R (Hsieh et al. 2005) were used for amplifying partial large-subunit ribosomal RNA (LSU), internal transcribed spacer (ITS), partial second-largest subunit of the RNA polymerase II (*rpb*2), β-tubulin (*tub*) and α-actin gene (Hsieh et al. 2005). The amplification conditions were carried out according to Liu et al. (2011) and Hsieh et al. (2005). Amplified products were examined and sent to the sequencing company, Sangon Biotech, Shanghai, China. The obtained sequences were checked, assembled and uploaded to GenBank.

### ﻿Sequence alignment and phylogenetic analyses

Following the NCBI BLAST results and literature (e.g. Jeewon et al. 2003; Senanayake et al. 2015), relevant sequences from all families of Xylariomycetidae were downloaded from GenBank for the phylogenetic analyses (Table 1). Sequences of each segment were aligned using MAFFT (http://mafft.cbrc.jp/alignment/server/index.html, Katoh and Standley 2019) and improved manually in BioEdit 7.2.3 (Hall 1999). The combined alignment of ITS, LSU, *rpb*2 and β-tubulin was concatenated from individual datasets. Ambiguously aligned areas of each gene region were excluded and gaps were treated as missing data. The ALTER (http://sing.ei.uvigo.es/ALTER/) phylogeny website tool was used to obtain the phylip file for RAxML analysis and the nexus file for Bayesian analysis (Glez-Peña et al. 2010). Phylogenetic trees were visualised using FigTree v.1.4.0. and processed using Adobe Photoshop CS6 software (Adobe Systems, USA). The alignment for the tree in this paper was uploaded on the website (https://treebase.org/) with submission ID 26613.

**Table 1.** Sequences used for phylogenetic analyses in this study.

Species	Strain number	Status	GenBank accession numbers				References
			ITS	LSU	*rpb2*	β-tubulin	
*Achaetomiummacrosporum*	CBS 532.94	–	KX976574	KX976699	KX976797	KX976915	Wang et al. (2016)
*Amphibambusabambusicola*	MFLUCC 11-0617	HT	KP744433	KP744474	N/A	N/A	Senanayake et al. (2015)
*Amphisphaeriaacericola*	MFLU 16-2479	HT	NR_171945	MK640424	N/A	N/A	Senanayake et al. (2019, submitted directly)
*Amphisphaeriathailandica*	MFLU 18-0794	HT	NR_168783	NG_068588	MK033640	MK033639	Samarakoon et al. (2019)
*Amphisphaeriaumbrina*	AFTOL-ID 1229	AF009805	N/A	FJ176863	FJ238348	N/A	Schoch (2008, submitted directly)
*Apiosporabambusae*	ICMP 6889	–	N/A	DQ368630	DQ368649	N/A	Tang et al. (2007)
*Apiosporahyphopodii*	MFLUCC 15-0003	HT	KR069110	KY356093	N/A	N/A	Dai et al. (2016)
*Apiosporasetosa*	ICMP 4207	–	N/A	DQ368631	DQ368650	DQ368620	Tang et al. (2007)
*Apiosporayunnana*	MFLUCC 15-0002	HT	KU940147	NG_057104	KU940177	MK291950	Dai et al. (2017)
*Arecophilaaustralis*	GZUCC0112	HT	MT742126	MT742133	N/A	MT741734	This study
*Arecophilaaustralis*	GZUCC0124	–	MT742125	MT742132	N/A	N/A	This study
*Arecophilabambusae*	HKUCC 4794	–	N/A	AF452038	N/A	N/A	Kang et al. (1999)
*Arecophilaclypeata*	GZUCC0110	HT	MT742129	MT742136	MT741732	N/A	This study
*Arecophilaclypeata*	GZUCC0127	–	MT742128	MT742135	N/A	N/A	This study
*Arecophilamiscanthi*	GZUCC0122	–	MT742127	MT742134	N/A	N/A	This study
*Arecophilamiscanthi*	MFLU 19-2333	HT	NR_171235	MK503827	N/A	N/A	Hyde et al. (2020)
*Arecophila* sp.	HKUCC 6487	–	N/A	AF452039	N/A	N/A	Jeewon et al. (2003)
*Apiosporayunnana*	MFLUCC 15-0002	HT	KU940147	NG_057104	KU940177	MK291950	Dai et al. (2017)
*Atrotorquataspartii*	MFLUCC 13-0444	HT	N/A	KP325443	N/A	N/A	Thambugala et al. (2015)
*Bagadiellalunata*	CBS 124762	HT	NR_132832	NG_058637	N/A	N/A	Cheewangkoon et al. (2009)
*Barrmaeliarappazii*	Cr2 = CBS 142771	HT	MF488989	MF488989	MF488998	MF489017	Voglmayr et al. (2018)
*Barrmaeliarhamnicola*	BR = CBS 142772	HT	MF488990	MF488990	MF488999	MF489018	Voglmayr et al. (2018)
*Bartaliniapondoensis*	CMW 31067	–	MH863602	MH875078	MH554904	MH554663	Vu et al. (2019)
*Beltraniapseudorhombica*	CBS 138003	HT	MH554124	NG_058667	MH555032	N/A	Liu et al. (2019)
*Beltraniarhombica*	CBS 123.58	T	MH857718	MH868082	MH554899	MH704631	Vu et al. (2019)
*Beltraniopsislongiconidiophora*	MFLUCC 17-2139	HT	NR_158353	NG_066200	N/A	N/A	Liu et al. (2017)
*Biscogniauxianummularia*	MUCL 51395	ET	KY610382	KT281894	KY624236	KX271241	Senanayake et al. (2015)
*Cainiaanthoxanthis*	MFLUCC 15-0539	HT	NR_138407	KR092777	N/A	N/A	Senanayake et al. (2015)
*Cainiagraminis*	CBS 136.62	–	MH858123	AF431949	N/A	N/A	Vu et al. (2019)
*Cainiagraminis*	MFLUCC 15-0540	–	KR092793	KR092781	N/A	N/A	Senanayake et al. (2015)
*Camilleaobularia*	ATCC 28093	–	KY610384	KY610429	KY624238	KX271243	Wendt et al. (2018)
*Castanediellaacaciae*	CBS 139896	HT	NR_137985	NG_067293	N/A	N/A	Crous et al. (2015)
*Castanediellacouratarii*	CBS 579.71	HT	NR_145250	NG_066249	N/A	N/A	Vu et al. (2019)
*Castanediellaeucalypticola*	CPC 26539	HT	KX228266	KX228317	N/A	KX228382	Crous et al. (2013)
*Chaetomiumelatum*	CBS 374.66	–	KC109758	KC109758	KF001820	KC109776	Wang et al. (2016)
*Ciferriascoseafluctuatimura*	MFLUCC 15-0541	HT	KR092789	KR092778	N/A	N/A	Senanayake et al. (2015)
*Ciferriascosearectimura*	MFLUCC 15-0542	HT	NR_153905	KR092776	N/A	N/A	Senanayake et al. (2015)
*Clypeophysalosporalatitans*	CBS 141463	ET	NR_153929	NG_058958	N/A	N/A	Giraldo et al. (2017)
*Coniocessiamaxima*	CBS 593.74	HT	NR_137751	MH878275	N/A	N/A	Vu et al. (2019)
*Coniocessianodulisporioides*	CBS 281.77	IT	MH861061	AJ875224	N/A	N/A	García et al. (2006)
*Creosphaeriasassafras*	STMA 14087	–	KY610411	KY610468	KY624265	KX271258	Wendt et al. (2018)
*Cylindriumaeruginosum*	CBS 693.83	–	KM231854	KM231734	KM232430	KM232124	Lombard et al (2014, submitted directly)
*Cylindriumgrande*	CBS 145655	HT	NR_165557	NG_068656	MK876481	MK876502	Crous et al. (2019)
*Cylindriumpurgamentum*	CPC 29580	HT	NR_155691	NG_067320	N/A	N/A	Koppel et al. (2017)
*Daldiniaconcentrica*	CBS 113277	–	AY616683	KT281895	KY624243	KC977274	Senanayake et al. (2015)
*Delonicicolasiamense*	MFLUCC 15-0670	HT	MF167586	NG_059172	MF158346	N/A	Perera et al. (2017)
*Diatrypepalmicola*	MFLUCC 11-0018	–	KP744439	KP744481	N/A	N/A	Liu et al. (2015)
*Diatrypewhitmanensis*	ATCC MYA-4417	–	FJ746656	FJ430587	N/A	N/A	Igo et al. (2009, direct submission)
*Entosordariaperfidiosa*	EPE = CBS 142773	ET	MF488993	MF488993	MF489003	MF489021	Voglmayr et al. (2018)
*Entosordariaquercina*	RQ = CBS 142774	HT	MF488994	MF488994	MF489004	MF489022	Voglmayr et al. (2018)
*Eutypaflavovirens*	MFLUCC 13-0625	–	KR092798	KR092774	N/A	N/A	Senanayake et al. (2015)
*Eutypalaevata*	CBS 291.87	–	HM164737	N/A	HM164805	HM164771	Trouillas and Gubler (2010)
*Eutypalata*	CBS 208.87	NT	MH862066	MH873755	KF453595	DQ006969	Vu et al. (2019)
*Furfurellanigrescens*	CBS 143622	HT	MK527844	MK527844	MK523275	MK523332	Voglmayr et al. (2019)
*Furfurellastromatica*	CBS 144409	HT	NR_164062	MK527846	MK523277	MK523334	Voglmayr et al. (2019)
*Graphostromaplatystomum*	AFTOL-ID 1249	HT	HG934115	DQ836906	DQ836893	HG934108	Zhang et al. (2006)
*Hyponectriabuxi*	UME 31430	–	-	AY083834	N/A	N/A	Smith et al. (2002, submitted directly)
*Hypoxylonfragiforme*	MUCL51264	ET	KM186294	KM186295	KM186296	KM186293	Daranagama et al. (2015)
*Iodosphaeriahonghensis*	MFLU 19-0719	HT	MK737501	MK722172	MK791287	N/A	Marasinghe et al. (2019)
*Iodosphaeriatongrenensis*	MFLU 15-0393	HT	KR095282	KR095283	N/A	N/A	Li et al. (2015)
*Jackrogersellamultiformis*	CBS 119016	ET	KC477234	KT281893	KY624290	KX271262	Wendt et al. (2018)
*Kretzschmariadeusta*	CBS 163.93	–	KC477237	KT281896	KY624227	KX271251	Senanayake et al. (2015)
*Lepteutypafuckelii*	CBS 140409	NT	NR_154123	KT949902	MK523280	MK523337	Jaklitsch et al. (2016)
*Leptosilliapistaciae*	CBS 128196	HT	NR_160064	MH798901	MH791334	MH791335	Voglmayr et al. (2019)
*Leptosilliawienkampii*	CBS 143630	ET	NR_164067	MK527865	MK523297	MK523353	Voglmayr et al. (2019)
*Longiappendisporachromolaenae*	MFLUCC 17-1485	HT	NR_169723	NG_068714	N/A	N/A	Mapook et al. (2020)
*Lopadostomaamericanum*	LG8	HT	KC774568	KC774568	KC774525	N/A	Jaklitsch et al. (2014)
*Lopadostomadryophilum*	LG21	ET	KC774570	KC774570	KC774526	MF489023	Jaklitsch et al. (2014)
*Lopadostomafagi*	LF1	HT	KC774575	KC774575	KC774531	N/A	Jaklitsch et al. (2014)
*Lopadostomaquercicola*	LG27	HT	KC774610	KC774610	KC774558	N/A	Jaklitsch et al. (2014)
*Lopadostomaturgidum*	LT2	ET	KC774618	KC774618	KC774563	MF489024	Jaklitsch et al. (2014)
*Melogrammacampylosporum*	MBU	–	JF440978	JF440978	N/A	N/A	Jaklitsch and Voglmayr (2012)
*Neophysalosporaeucalypti*	CBS 111123	–	KP031107	KP031109	N/A	N/A	Crous et al. (2014)
*Neophysalosporaeucalypti*	CBS 138864	HT	KP004462	MH878627	N/A	N/A	Crous et al. (2014)
*Oxydothismetroxylicola*	MFLUCC 15-0281	HT	KY206774	KY206763	KY206781	N/A	Konta et al. (2016)
*Oxydothispalmicola*	MFLUCC 15-0806	HT	KY206776	KY206765	KY206782	N/A	Konta et al. (2016)
*Oxydothisphoenicis*	MFLUCC 18-0269	HT	MK088065	MK088061	N/A	N/A	Hyde et al. (2020)
*Phlogicylindriumuniforme*	CBS 131312	HT	JQ044426	JQ044445	MH554910	MH704634	Crous et al. (2011)
*Podosordariatulasnei*	CBS 128.80	–	KT281902	KT281897	N/A	N/A	Senanayake et al. (2015)
*Poroniapunctata*	CBS 656.78	HT	KT281904	KY610496	KY624278	KX271281	Wendt et al. (2018)
*Pseudomassariachondrospora*	MFLUCC 15-0545	–	KR092790	KR092779	N/A	N/A	Senanayake et al. (2015)
*Pseudomassariasepincoliformis*	CBS 129022	–	JF440984	JF440984	N/A	N/A	Jaklitsch and Voglmayr (2012)
*Pseudosporidesmiumknawiae*	CBS 123529	HT	MH863299	MH874823	N/A	N/A	Crous et al. (2017, submitted directly)
*Pseudosporidesmiumlambertiae*	CBS 143169	HT	NR_156656	NG_058506	N/A	N/A	Crous et al. (2017)
*Pseudotruncatellaarezzoensis*	MFLUCC 14-0988	HT	NR_157489	NG_070426	N/A	N/A	Perera et al. (2018)
*Pseudotruncatellabolusanthi*	CBS 145532	HT	NR_165575	MK876448	N/A	N/A	Crous et al. (2019)
*Robillardaroystoneae*	CBS 115445	HT	NR_145251	NG_069287	MH554880	KR873317	Liu et al. (2019)
*Sarcoxyloncompunctum*	CBS 359.61	–	MH858083	KY610462	KY624230	KX271255	Wendt et al.(2018)
*Seiridiummarginatum*	CBS 140403	ET	NR_156602	MH554223	LT853149	MT853249	Liu et al. (2019)
*Seynesiaerumpens*	SMH 1291	–	N/A	AF279410	AY641073	N/A	Bhattacharya et al. (2000)
*Sordariafimicola*	CBS 723.96	–	MH862606	MH874231	DQ368647	DQ368618	Vu et al. (2019)
*Sporocadusrotundatus*	CBS 616.83	HT	NR_161091	NG_069584	MH554974	MH554737	Liu et al. (2019)
*Subsessilaturbinata*	MFLUCC 15-0831	HT	NR_148122	NG_059724	N/A	N/A	Lin et al. (2017)
*Vialaeainsculpta*	DAOM 240257	–	JX139726	JX139726	N/A	N/A	Hambleton et al. (2010, submitted directly)
*Vialaeamangiferae*	MFLUCC 12-0808	HT	NR_171903	NG_073594	N/A	N/A	Senanayake et al. (2021, submitted directly)
*Vialaeaminutella*	BRIP 56959	–	KC181926	KC181924	N/A	N/A	McTaggart et al. (2013)
*Xyladictyochaetalusitanica*	CBS 143502	–	MH107926	MH107972	N/A	MH108053	Crous et al. (2013)
*Xylariahypoxylon*	CBS 122620	ET	KY610407	KY610495	KY624231	KX271279	Wendt et al. (2018)
*Xylariaobovata*	MFLUCC 13-0115	–	KR049088	KR049089	N/A	N/A	Wendt et al. (2018)
*Xylariapolymorpha*	MUCL 49884	–	KY610408	KT281899	KY624288	KX271280	Wendt et al. (2018)

Note. Type specimens are labelled with HT (holotype), ET (epitype) and IT (isotype), T (Type). N/A: not available.

Maximum likelihood (ML) analysis was performed on the CIPRES Science Gateway v.3.3 (http://www.phylo.org/portal2; Miller et al. 2010) using RAxML v.8.2.8 as part of the ‘RAxML-HPC BlackBox’ tool (Stamatakis et al. 2008). All free model parameters were estimated by RAxML with ML estimates of 25 per-site rate categories. GTRGAMMA + I model was chosen for RAxML, based on the result of MrModeltest 2.2. The best-scoring tree was selected with a final likelihood value of –10720.566919.

A Bayesian analysis (BY) was performed using MrBayes v.3.2.2 (Ronquist et al. 2012). The best-fit model was selected with MrModeltest 2.2 (Nylander 2004). Posterior probabilities (PP) (Rannala and Yang 1996) were determined by Markov Chain Monte Carlo sampling (MCMC) (Ronquist and Huelsenbeck 2003). Six simultaneous Markov chains were initially run for 30 × 10^6^ generations and for every 1000^th^ generation, a tree was sampled (resulting in 30,000 total trees). The MCMC heated chain was set with a ‘temperature’ value of 0.15. All sampled topologies beneath the asymptote (20%) were discarded. The remaining 24,000 trees were used to calculate the posterior probability (PP) values in the majority rule consensus tree (Liu et al. 2011).

## ﻿Results

### ﻿Phylogenetic analyses

**Figure 1.**
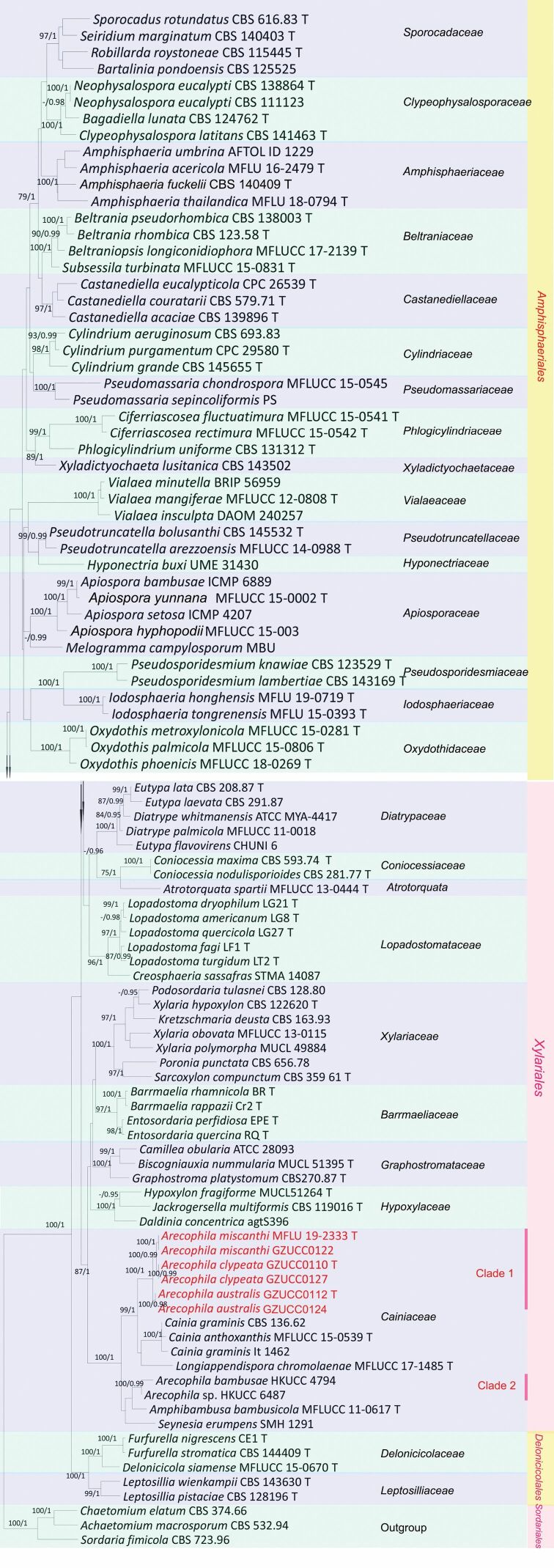
Phylogenetic tree, based on a combined ITS, LSU, *rpb*2 and β-tubulin gene dataset. Numbers close to each node represent Maximum Likelihood bootstrap values (≥ 75%) and Bayesian posterior probabilities (≥ 0.95). The hyphen (“–”) means a value lower than 75% (BS) or 0.95 (PP). New taxa are marked in red. Type materials are marked with T after the strains. The tree is rooted to *Achaetomiummacrosporum* (CBS 532.94), *Chaetomiumelatum* (CBS 374.66) and *Sordariafimicola* (CBS 723.96).

The resulted trees from ML and BY were similar in topology. *Cainiaceae* is a monophyletic group (Fig. 1) with 100%/1.00 (PP/BS) support. *Arecophila* species form two clades. Clade 1 consists of *A.miscanthi* (≡ *Alishanicamiscanthi*), *A.clypeata* and *A.australis*, with high statistical support (100%/1.00 PP). In Clade 2, *A.bambusae* (HKUCC 4794) and *Arecophila* sp. (HKUCC 6487) display a close relationship with *Amphibambusabambusicola*.

### ﻿Taxonomy

#### ﻿ Arecophila

Taxon classificationFungiXylarialesAmphisphaeriaceae

K.D. Hyde, Nova Hedwigia 63(1–2): 82 (1996)

F28DD8D5-5910-5F1B-BF13-11C2653DC3B0

 27653

≡ Alishanica Karun., C.H. Kuo & K.D. Hyde, in Hyde et al., Mycosphere 11(1): 460 (2020)

##### Sexual morph.

*Ascomata* immersed, raised, blackened areas on the host surface, a central erumpent, short, cone-shaped or umbilicate papilla, subglobose to lenticular in vertical section. Clypeus present or not, comprising host cells and intracellular brown hyphae. *Peridium* comprising several layers of angular cells. *Paraphyses* hypha-like, filamentous, septate, hyaline. *Asci* 8-spored, unitunicate, cylindrical, with an apical ring bluing in Melzer’s reagent or not. *Ascospores* ellipsoidal, 2-celled, constricted at the septum, brown, with longitudinal striations or a verrucose wall and surrounded by a wide mucilaginous sheath (Hyde 1996).

##### Asexual morph.

Undetermined.

#### ﻿ Arecophila australis

Taxon classificationFungiXylarialesAmphisphaeriaceae

Q.R. Li, J.C. Kang & K.D. Hyde sp. nov.

BFDFCDD6-E7CD-5553-AB3D-217FDC12ED2B

 836166

Fig. 2

##### Diagnosis.

*Arecophilaaustralis* differs from similar species by its dimension of ascospores (22.5–29 × 8–11 µm) covered by striations and ascomata with a disc area surrounding the ostioles.

**Figure 2.**
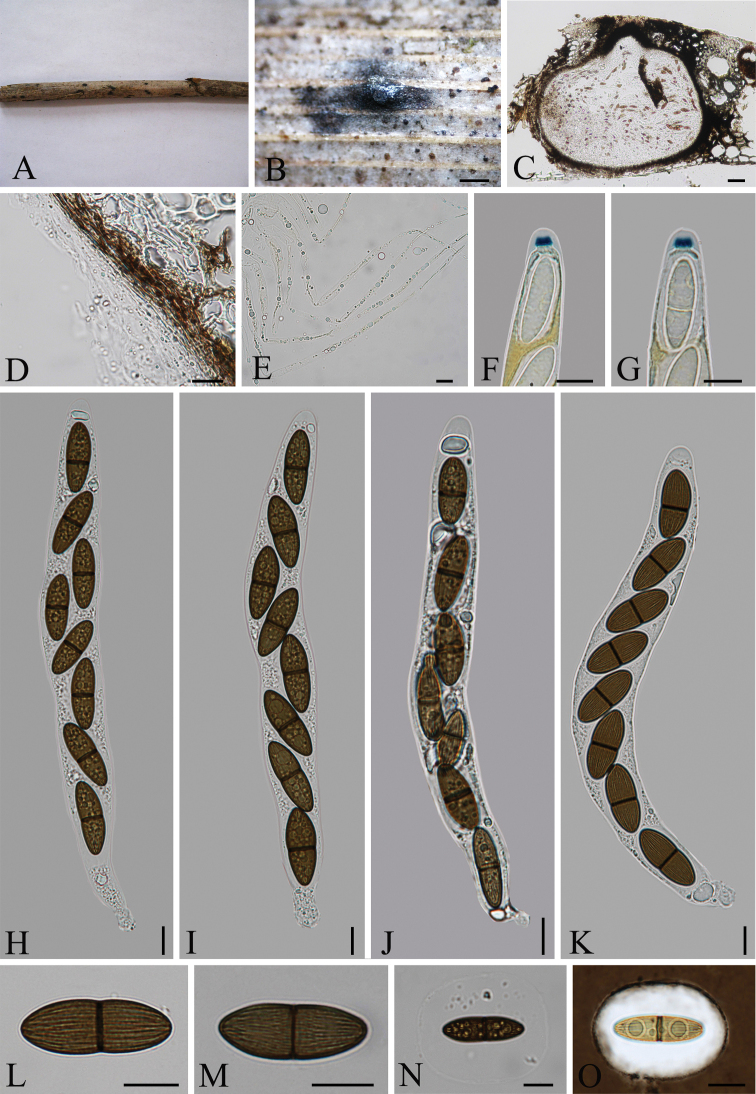
*Arecophilaaustralis* (holotype) **A** material **B** ascoma on the surface of host **C** section of ascoma **D** peridium **E** paraphyses **F, G** ascus apex with a J+, apical ring (stained in Melzer’s reagent) **H–K** asci with ascospores **L–O** ascospores surrounded by a wide mucilaginous sheath (O stained in India ink). Scale bars: 300 μm (**B**); 50 μm (**C**); 5 μm (**D–O**).

***Holotype*.** China, Guizhou Province, Guiyang City, Forest Park of Guiyang (26°32'55"N, 106°45'25"E), on dead culm of *Phragmitesaustralis* (Cav.) Steud., 15 March 2014, Q.R. Li, GZ58 (GZUH0112, ***holotype***, **ex-type**: GZUCC0112; GACP QR0152, ***isotype***).

##### Additional sequences.

ACT: MT741737

##### Etymology.

In reference to the host, *Phragmitesaustralis* (Cav.) Steud. *australis*

##### Description.

Saprobic on dead culm of gramineous host. **Sexual morph**: *Ascomata* 420–560 × 290–380 µm (*x̄*= 495 × 325 µm, n = 10), immersed under a clypeus, solitary, slightly raised, blackened, dome-shaped areas, scattered or gregarious, globose to subglobose, with a central, erumpent, cone-shaped papilla in vertical section. Clypeus black, comprising host cells and intracellular brown hyphae. *Ostioles* papillate, black. *Peridium* 15–25 µm (*x̄*= 21 µm, n = 15) wide, comprising several layers, outer layer brown, thick-walled angular cells, inner layer hyaline. *Paraphyses* 3.3–5 μm (*x̄*= 3.5 µm, n = 15) wide, hyaline, unbranched, septate. *Asci* 140–230 × 15.5–24 µm (*x̄*= 183.5 × 19 µm, n = 30), 8-spored, unitunicate, long-cylindrical, short-pedicellate, apically rounded, with a 4–5 × 2.5–3 μm (*x̄*= 4.5 × 2.7 μm, n = 20), trapezoidal, J+, apical ring. *Ascospores* 22.5–29 × 8–11 µm (*x̄*= 25.5 × 9 µm, n = 30), overlapping uniseriate, 2-celled, light brown to brown, equilateral ellipsoidal, constricted at the septum, longitudinal with sulcate striations, along the entire spore length, surrounded by a mucilaginous sheath, lacking germ slits and appendages. **Asexual morph**: undetermined.

##### Culture characteristics.

Colonies on PDA, reached 3 cm diam. after one week at 25 °C, white, cottony, flat, low, dense, with slightly wavy margin.

##### Known distribution.

China

##### Additional material examined.

China, Guizhou Province, Guiyang City, Leigongshan National Nature Reserve (26°21'39"N, 108°9'59"E), on dead culm of an unidentified gramineous plant, 13 June 2015, Q.R. Li, GY67 (GACP QR0124, GZUH 0136; living cultures, GZUCC0124).

##### Notes.

*Arecophilaaustralis* resembles *A.serrulata* (Ellis & Martin) K.D. Hyde and *A.calamicola* K.D. Hyde (Hyde 1996). However, *A.serrulata* has white ring surrounding ostioles of ascomata, narrower ascospores (17–26 × 7–9.5 µm vs. 22.5–29 × 8–11 µm), smaller asci and apical ring (3.2 × 2.4 µm vs. 4.5 × 2.7 μm) compared to *A.australis* (Hyde 1996). *Arecophilacalamicola* differs from *A.australis* in lacking clypeus, ascospores covered by verrucose ornamentation and surrounding by a mucilaginous sheath attached at the poles. Molecular phylogeny, based on combined ITS, LSU, *rpb*2 and β-tubulin sequences, shows that *A.australis* clusters as a distinctive clade in *Arecophila* (Clade 1). Based on its distinct morphology and phylogeny, *A.australis* is introduced as a new species. Here, we need to explain the name of *A.serrulata*. Although Index Fungorum (02/07/2022) shows that the current name of *A.serrulata* is *Roussoellaserrulata* (Ellis & G. Martin) K.D. Hyde & Aptroot, we have not found relevant literature. Hyde (1996) renamed *Didymosphaeriaserrulota* Eltis & G. Martin and *Roussoellaserrulata* as synonyms of *A.serrulata* (Ellis & G. Martin) K.D. Hyde. *Arecophilaserrulata* was erected with the unitunicate asci with a blue-staining ring (Hyde 1996) which is clearly inconsistent with the morphological features of *Roussoella* Sacc. Therefore, we still compare with the original description of *A.serrulata* in this article.

#### ﻿ Arecophila clypeata

Taxon classificationFungiXylarialesAmphisphaeriaceae

Q.R. Li, J.C. Kang & K.D. Hyde sp. nov.

B31AA394-95A5-5A80-9A0D-796D0C51AC6E

 836167

Fig. 3

##### Diagnosis.

*Arecophilaclypeata* differs from similar species by its ascomata with clypeus and ascospores (18.5–22.5 × 6.5–9 µm).

***Holotype*.** China, Yunnan Province, Kunming City, Kunming Botanical Garden (25°8'51"N, 102°44'57"E), on dead culm of gramineous plant, 20 March 2014, Q.R. Li, kib21 (***holotype***: GZUH0110; ***isotype***: GACP QR0173; **ex-type** living cultures: GZUCC0110).

**Figure 3.**
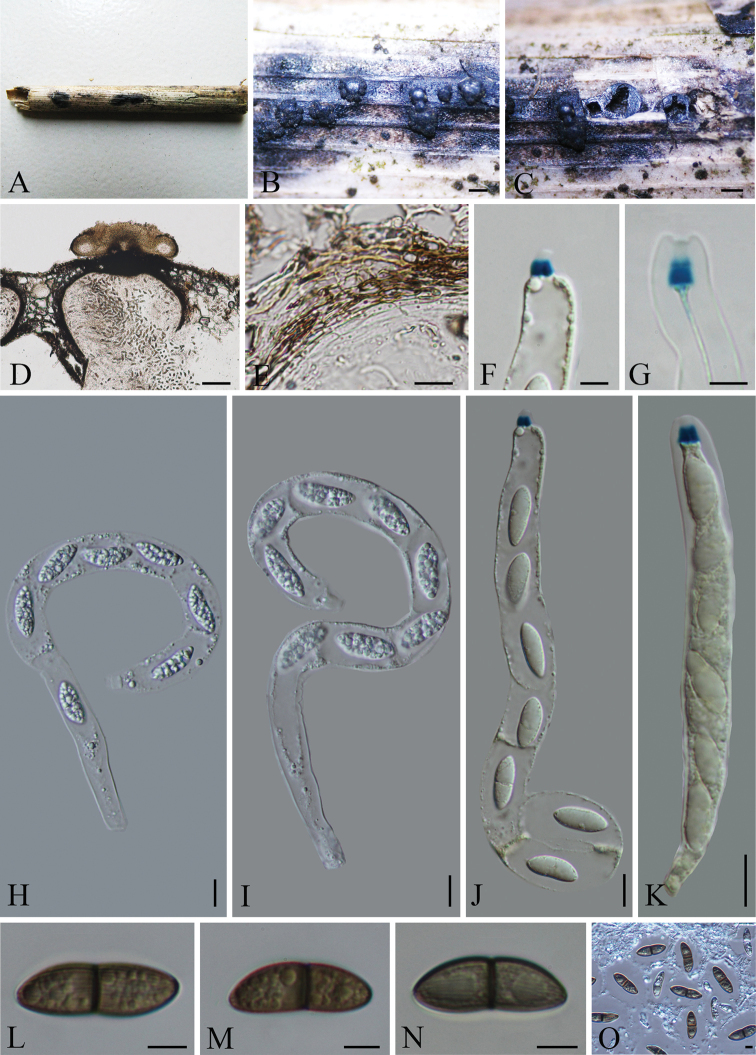
*Arecophilaclypeata* (holotype) **A** material **B** ascomata on the surface of host **C, D** section of ascomata **E** peridium **F, G** ascus apex with a J+, apical ring (stained in Melzer’s reagent) **H–K** asci with ascospores **L–O** ascospores. Scale bars: 500 μm (**B, C**); 100 μm (**D**); 10 μm (**E, H–K**); 5 μm (**F, G, L–O**).

##### Etymology.

In reference to the clypeus.

##### Description.

Saprobic on dead stem of a gramineous. **Sexual morph**: *Ascomata* 367–448 × 278–363 µm (*x̄*= 403 × 323 µm, n = 8), immersed under a black clypeus, solitary, slightly raised, dome-shaped areas, scattered or gregarious, subglobose to globose, with a central, erumpent, cone-shaped papilla, in vertical section. Ostioles papillate on the centre, black. *Peridium* 15–30 µm (*x̄*= 25 µm, n = 10) wide, comprising several layers, outer layer brown, thick-walled angular cells, inner layer hyaline. *Paraphyses* 3–5 μm (*x̄*= 4 µm, n =15) wide, hyaline, unbranched, septate. *Asci* 180–245 × 10.5–14.5 µm (*x̄*= 215.5 × 12 µm, n=20), 8-spored, unitunicate, long-cylindrical, short-pedicellate, apically rounded, with a square-shaped, J+, apical ring, 3–4 × 3–4 μm. *Ascospores* 18.5–22.5 × 6.5–9 µm (*x̄*= 20.5 × 7.5 µm, n = 30), overlapping uniseriate, 2-celled, light brown to brown, equilateral ellipsoidal, constricted at the septum, longitudinal, sulcate along the entire spore length, faint, surrounded by a mucilaginous sheath, lacking germ slits and appendages. **Asexual morph**: undetermined.

##### Culture characteristics.

Colonies on PDA, reached 3 cm diam. after one week at 25 °C, white, cottony, flat, low, dense, with slightly wavy margin; fructifications were not observed in culture.

##### Known distribution.

China

##### Additional material examined.

China, Guizhou Province, Buyi and Miao Autonomous Prefecture in southern Guizhou Province, Maolan National Nature Reserve (25°17'17"N, 107°59'1"E), on dead culm of an unidentified gramineous plant, 12 June 2015, Q.R. Li, GZ120 (GACP QR0129; GZUH0127; living cultures, GZUCC0127).

##### Additional sequences.

ACT: MT741737

##### Notes.

*Arecophilaclypeata* has long and weakly striate ascospores similar to *A.coronata* (Rehm) Umali & K.D. Hyde, *A.serrulata* (Ellis & G. Martin) K.D. Hyde and *A.bambusae* (Hyde 1996; Umali et al. 1999). However, *A.coronata* does not have a prominent clypeus and has longer and fusiform ascospores. *Arecophilaclypeata* differs from *A.serrulata* by the ascomata without a central papilla surrounded by a circle of white tissue, further in having ascospores with wide sheaths (Hyde 1996). *Arecophilaclypeata* is similar to *A.bambusae* which, however, has narrower ascospores (19–22.5 × 5.5–7 µm) covered by the strong striations and has ascomata without a central papilla surrounded by a black corolla protuberance (Umali et al. 1999).

#### ﻿ Arecophila gulubiicola

Taxon classificationFungiXylarialesAmphisphaeriaceae

K.D. Hyde, Nova Hedwigia 63(1–2): 91 (1996)

E533ED2A-0E76-55FA-A447-14E32DF15B0E

 416041

Fig. 4

##### Description.

*Saprobic* on dead trunk of *Gulubiacostata* (Becc.) Becc. **Sexual morph**: *Ascomata* 290–400 × 140–190 μm (*x̄* = 336 × 167 µm, n = 8), immersed under a clypeus, solitary or clustered, in vertical section, lenticular, with a central ostiole. *Clypeus* raised, oval, blackened areas on the host surface, dome-shaped, well-developed and black. *Peridium* 25–35 μm wide, dense, compressed layers of brown-walled, *angular* cells, tightly adhered to the host tissues. *Paraphyses* 2–2.5 μm wide, filamentous, hyaline, septate, branched, tapering distally. *Asci* 107–145 × 11–13.5 μm (*x̄* = 114.3 × 12.4 μm, n = 15), 8-spored, unitunicate, cylindrical, short-pedicellate, apically rounded, wedge-shaped, J+, subapical ring, 3–4 × 1–2 μm (*x̄* =3.5 × 1.5 μm, n = 15). *Ascospores* 14.5–18.5 × 6–9 μm (*x̄* = 17.4 × 6.5 μm, n = 25), overlapping uniseriate, ellipsoidal, brown, 2-celled, septate at the centre, constricted at the septum, longitudinal, sulcate striations along the entire spore length, surrounded by a mucilaginous sheath. **Asexual morph**: Undetermined.

**Figure 4.**
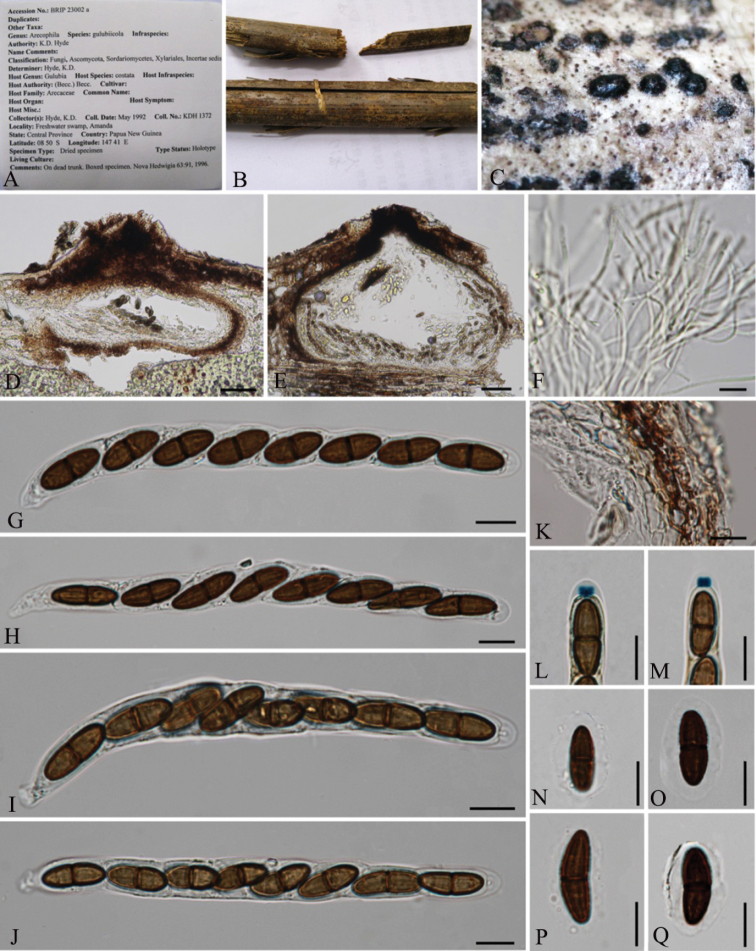
*Arecophilagulubiicola* (BRIP 23002a, holotype) **A, B** herbarium material with label **C** ascomata on the host **D, E** sections of ascomata **F** paraphyses **G–J** asci **K** peridium **L, M** wedge-shaped, J+ apical ring bluing in Melzer’s reagent **N–Q** ascospores. Scale bars: 50 μm (**D, E**); 5 μm (**F–Q**).

##### Material examined.

Papua New Guinea, Central Province, 08°30'00"N, 147°24'35"E, on dead trunk of *G.costate* (Becc.) Becc. (*Arecaceae*), May 1992, K.D. Hyde, (BRIP 23002a, ***holotype***).

##### Notes.

*Arecophilagulubiicola* has deeply immersed, subglobose to lenticular ascomata with a small or lacking clypeus, cylindrical, short-pedicellate asci with a wedge-shaped, conical, apical ring and ellipsoidal, brown ascospores with wall striations and surrounded by a mucilaginous sheath (Hyde 1996). *Alishanica* has been introduced as a monospecific genus with the type species *Al.miscanthi* Karun. et al. on dead sheaths of *Miscanthussinensis* (Poaceae) from Taiwan (Hyde et al. 2020). We re-examined both *A.gulubiicola* and *Al.miscanthi* herbarium specimens and observed that they are congeneric. *Alishanicamiscanthi* has characters that immersed ascoma under a clypeus, unitunicate, cylindrical asci with a J+ apical ring and brown, 2-celled ascospores with longitudinal wall striations and a mucilaginous sheath which are consistent with the generic characteristics of *Arecophila*. The phylogeny of *Al.miscanthi* was mainly considered by the *A.bambusae* (HKUCC 4794) sequences (Hyde et al. 2020). However, HKUCC 4794 is not the type material of *Arecophila* and cannot be used to represent *Arecophila*. In our phylogeny, HKUCC 4794 forms a distinct clade (Fig. 1; Clade 2) from the *Arecophila* representing the clade. Based on morphology and phylogeny, we synonymise *Alishanica* under *Arecophila* and *Al.miscanthi* is accepted as an *Arecophila* species. Furthermore, *A.bambusae* needs to be recollected and provided with the phylogenetic affinity in future studies.

#### ﻿ Arecophila miscanthi

Taxon classificationFungiXylarialesAmphisphaeriaceae

(Karun., C.H. Kuo & K.D. Hyde) Q.R Li & J.C. Kang comb. nov.

555587DE-0701-5A9E-B0BC-CD2BF216FBA8

 839706

≡ Alishanicamiscanthi Karun., C.H. Kuo & K.D. Hyde [as ‘miscanthii’], in Hyde et al., Mycosphere 11(1): 461 (2020)

##### Description

**(MFLU 19-2333).***Saprobic* on dead sheaths of *Miscanthussinensis* (Poaceae). **Sexual morph**: *Ascomata* 272–277 × 283–296 µm (*x̄* = 275 × 291.5 µm, n = 8), immersed beneath blackened aggregated *clypeus* of the surface of dead sheath, loosely aggregated or rarely solitary; dark brown to black, globose to subglobose, slightly depressed, uniloculate. *Ostiole* 92–110 μm long, 52–56 μm diameter (*x̄* = 101 × 54 μm, n = 5), centrally erumpent, with periphyses, surrounded by distinct shiny black flanges, the tissue spreading down along the papilla. *Peridium* 51–60 μm wide, comprising 4–5 cell layers of thin-walled, brown cells of *textura angularis*, inwardly lighter. *Paraphyses* filamentous, distinctly septate, embedded in a hyaline gelatinous matrix. *Asci* 147–189 × 10–13 μm (*x̄* = 167 × 11 μm, n = 30), 8-spored, unitunicate, cylindrical, short pedicellate, slightly truncate at the apex, with a wedge-shaped J+, subapical ring, 3.5–4 µm broad, 2–2.5 µm high. *Ascospores* 20–24 × 6–8 μm (*x̄* = 22 × 7 μm, n = 40), overlapping, uniseriate, ellipsoidal, slightly tapering at the ends, equally 2-celled and guttulate at both cells, constricted at the septum, brown with striations, surrounded by a thick, hyaline mucilaginous sheath, subglobose, parallel to the margin of the spore. **Asexual morph**: Undetermined.

##### Material examined.

China, Taiwan, Chiayi Province, Ali Mountain, Kwang Hwa, on dead sheaths of *Miscanthussinensis* (Poaceae), 5 May 2018, A. Karunarathna, AKTW 44 (**MFLU 19-2333**, ***holotype***)

##### Additional material.

China, Yunnan Province, Kunming City, Kunming Botanical Garden (25°8'45"N, 102°44'59"E), on dead culm of monocotyledon, 20 March 2014, Q.R. Li, GZ43 (GZUH0122, GACP QR0201; living cultures, GZUCC0122).

##### Note.

**Figure 5.**
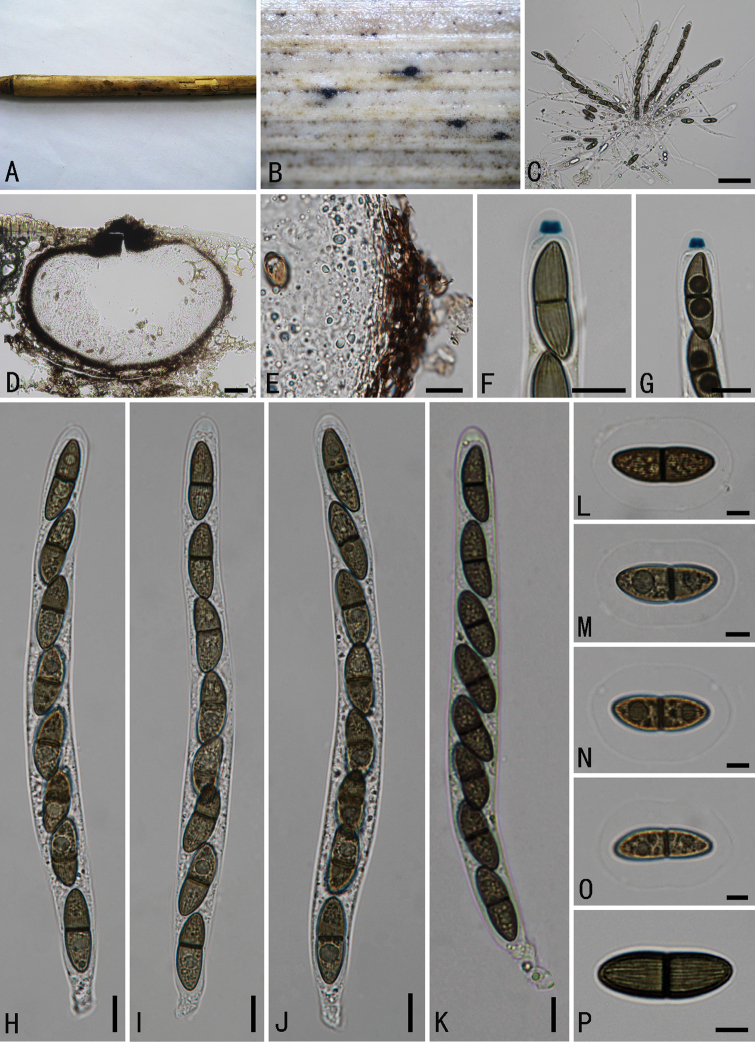
*Arecophilamiscanthi* (GZUH0122) **A, B** ascomata on the surface of host **C** paraphyses and asci **D** section of ascoma **E** peridium **F, G** apical rings **H–K** asci with ascospores **M–P** ascospores. Scale bars: 50 μm (**C, D**); 10 μm (**E, F–K**); 5 μm (**L–P**).

The characteristics of the holotype specimen *Arecophilamiscanthi* (≡ *Alishanicamiscanthi*) were revised, re-measured and described. *Alishanicamiscanthi* is similar to *A.muroiana* and *A.serrulata* (Wang et al. 2004, Hyde et al. 2020). However, no clypeus was observed for *A.muroiana*. *Arecophilaserrulata* has larger ascomata (480–560 × 280–320 μm) with a central papilla surrounded by a circle of white tissue (Hyde 1996) which differs from those of *A.miscanthi*. One new collection (GZUH0122, Fig. 5) shows the same traits of *A.miscanthi* (MFLU 19-2333) in having immersed ascomata with clypeus, a wedge-shaped J+, ascus subapical ring, same dimensions of ascospores and here we provide it as a new geographical record from China.

## ﻿Discussion

*Arecophila* shares similar morphology to *Atrotorquata*, *Cainia* and *Seynesia* in having immersed ascomata and 2-celled ascospores (Hyde 1996). *Arecophila*, *Atrotorquata*, *Cainia* and *Seynesia* are accepted in *Cainiaceae* with newly-introduced genera, such as *Amphibambusa* and *Longiappendispora* (Mapook et al. 2020). *Cainia* has similar characteristics to *Arecophila* in its occurrence on monocotyledons, having asci with J+, apical rings and brown 2-celled ascospores (Kohlmeyer and Volkmann-Kohlmeyer 1993). The ascospores of *Cainia* are provided with several longitudinal germ slits and differ from those of *Arecophila*, where the ascospores are provided with ridges or a verrucose wall and lack germ slits. *Seynesia* produces ascospores that are smooth-walled and surrounded by mucilaginous sheaths that are drawn out at the poles with germ slits, which differ from *Arecophila* (Hyde 1995). *Amphibambusa* possesses hyaline ascospores pointed at both ends, which differs from that of *Arecophila* (Liu et al. 2015). The phylogenetic tree (Fig. 1) displays that *Arecophilamiscanthi* (≡ *Alishanicamiscanthi*) clusters in the *Arecophila* group with high support values (100%/1.00 PP). *Longiappendispora* possesses ascospores with longitudinal striations and bristle-like polar appendages at both ends, without a gelatinous sheath, which differentiates it from other genera in *Cainiaceae*. Ascospores of *Atrotorquata* are provided with several longitudinal germ slits and differ from those of *Arecophila* (Kohlmeyer and Volkmann-Kohlmeyer 1993). At present, 16 *Arecophila* species have been described and a summary of each species are given in the Table 2.

**Table 2.** Synopsis of the species of *Arecophila*.

Species	Host	Clypeus	Ascomata	Asci	Ascal ring	Ascospores	Distribution
*A.australis*	*Phragmitesaustralis*	Present	420–560 × 290–380 µm, globose to subglobose	140–230 × 15.5–24 µm	4–5 × 2.5–3 μm, trapezoidal, J+	22.5–29 × 8–11 µm, wall striate, mucilaginous sheath	China (Guizhou)
*A.bambusae*	*Bambusa* sp.	Absent	500–560 × 294–350 µm, globose to subglobose	132.5–140 × 7.5–8 µm	2.5–3 µm in diam., ca. 2.5 µm high, wedge-shaped, J+	19–22.5 × 5.5–7 µm, slightly tapering at the ends, wall striate, mucilaginous sheath	Hong Kong
*A.calamicola*	*Calamus* sp.	Absent	520 × 390 µm, subglobose	160–190 × 14–20 µm	4-4.8 µm diam., 3.2-4 µm high, wedge-shaped, J+	24–33 × 5.5–9 µm, wall striate, verrucose, mucilaginous sheath	Brunel, Indonesia
*A.chamaeropis*	*Chamaeropshumilis*	Minute	400–700 × 300–400 µm, subglobose	150–190 × 9–10 µm	3.5–4.5 diam., 1.5–2 µm high, wedge-shaped, J+	15–23 × 5.5–7 µm, wall striate, covered by pronounced verrucose ornamentation, mucilaginous sheath	Spain
*A.coronata*	*Gigantochloascribneriana*, *Bambusa* sp.	Present	90–100 × 42–105 µm, subglobose or ellipsoidal	132.5–157.5 × 7.5–9 µm	3.5–4 µm in diam., 2–2.5 µm high, wedge-shaped, J+, with a faint canal leading to the apex.	29–31 × 5–5.5 µm, wall faint striate, mucilaginous sheath	Philippines, Hong Kong
*A.clypeata*	A unknown gramineous plant	Present	367‒448 × 278‒363 µm, subglobose to globose	180–245 × 10.5–14.5 µm	3–4 × 3–4 μm, square-shaped, J+	18.5–22.5 × 6.5–9 µm, wall striate, mucilaginous sheath	China (Guizhou)
*A.deutziae*	*Deutziaestamineae*	Absent	400–600 µm diam., globose	180–240 × 16–19 µm	3.5–4. 5 µm diam., 1.5–2 µm high, wedge-shaped, J+	26–32 × 11–13 µm, wall striate	India
*A.eugeissonae*	*Eugeissonatristis*	Absent	460–520 × 180–260 µm, Subglobose or ellipsoidal	175–220 × 11–16.5 µm	3–4 µm diam., 1.5–2.0 µm high, discoid, J+	25-40 × 6.5–9 µm, wall weakly striate, verrucose, mucilaginous sheath	Malaysia
*A.foveata*	*Nolinae* sp.	Present	300–400 ×400–500 µm, globose or ovoid	130–150 × 14–15 µm	3–4 µm wide, 4–5 µm high, tubular, J+	16–20 × 8–10 µm, wall striate, foveate, surface aspect of numerous warts	USA
*A.gulubiicola*	*Gulubiacostate*	Present	290–400 × 140–190 μm, subglobose or lenticular	107–145 × 11–13.5 μm	3.2–4 µm diam., 2.4–3.2 µm high, cylindrical, J+	14.5–18.5 × 6–9 μm with a minutely verrucose wall, mucilaginous sheath	Papua New Guinea
*A.miscanthi*	*Miscanthussinensis*	Present	283–296 × 272–277 µm, globose to subglobose	147–189 × 10–13 μm	3.5–4 µm broad, 2–2.5 µm high, wedge-shaped, J+	20–24 × 6–8 μm, wall striate, mucilaginous sheath.	China (Taiwan, Yunnan)
*A.muroiana*	*Phyllostachysbambusoides*	Absent	350–460 × 320–400 µm, globose	125–165 × 10–12 µm	3.5–4 µm diam., 2–2.5 µm high, wedge-shaped, J+	20–25 × 6–7.5 µm, wall finely striate, mucilaginous sheath	Japan
*A.notabilis*	*Calamus*, *Bamboo*	Present	400 × 360 µm, subglobose	180–220 × 11–14 µm	4–4.45 µm diam., 3–4.5 µm high, wedge-shaped, J+	20–26 × 6–8 µm, wall striate, finely verrucose, mucilaginous sheath	Brunei, Hong Kong, Indonesia
*A.nypae*	*Nypafruticans*	Absent	400–500 µm diam., subglobose	140–205 × 11–13 µm	4.5 µm diam., 2.5–4 µm high, wedge-shaped, J+	19–26 × 7–8 µm, wall striate, mucilaginous sheath	Malaysia
*A.saccharicola*	*Sacchariofficinarum*	Absent	420–525 × 350–420 µm high	140–16 × 7–10 µm	Not blued by Melzer’s reagent	20–24 × 6–8 µm, wall smooth or striated	Jamaica
*A.serrulata*	*Korthalsia* sp., *Sabal* sp., *Serenoa* sp.	Present	480–560 × 280–320 µm, conical with flattened base	110–112 × 10–12 µm,	3.2 µm diam., 2.4 µm high, wedge-shaped, J+	17–26 × 7–9.5 µm, wall striate, mucilaginous sheath	Brunei, USA, Florida

The combined ITS, LSU, *rpb*2 and β-tubulin phylogeny (Fig. 1) showed two clades of *Arecophila* as Clade 1 and Clade 2. The *Arecophila* differs from *Amphibambusa* and *Cainia* (see above). The sequence from the holotype of *Atrotorquataspartii* is noticeably clustered with *Coniocessia* spp. in *Coniocessiaceae* (Fig. 1). However, *Atrotorquataspartii* showed a close affinity with *Cainia* spp. in *Cainiaceae*, based on analysis of the combined LSU and ITS sequence alignment in Senanayake et al. (2015). *Atrotorquata* has similar characteristics to *Arecophila* and other genera of *Cainiaceae* (Hyde 1996). Hence, there should be more evidence to reassess *Atrotorquata* in the future. The unitunicate asci with a J+ apical ring in Melzer’s regent and brown ascospores covered with longitudinal wall striations, without germ slits can clearly distinguish *Arecophila* from its similar genera. In addition, a table including synopsis of the species of *Arecophila* is provided.

## Supplementary Material

XML Treatment for Arecophila

XML Treatment for Arecophila australis

XML Treatment for Arecophila clypeata

XML Treatment for Arecophila gulubiicola

XML Treatment for Arecophila miscanthi
